# An approach to increase the success rate of cultivation of soil bacteria based on fluorescence-activated cell sorting

**DOI:** 10.1371/journal.pone.0237748

**Published:** 2020-08-31

**Authors:** Laura Espina

**Affiliations:** Department of Medical Microbiology, Cardiff University, Cardiff, United Kingdom; Stazione Zoologica Anton Dohrn, ITALY

## Abstract

Soil microbiota are considered a source of undiscovered bioactive compounds, yet cultivation of most bacteria within a sample remains generally unsuccessful. Two main reasons behind the unculturability of bacteria are the presence of cells in a viable but not culturable state (such as dormant cells) and the failure to provide the necessary growth requirements *in vitro* (leading to the classification of some bacterial taxa as yet-to-be-cultured). The present work focuses on the development of a single procedure that helps distinguish between both phenomena of unculturability based on viability staining coupled with flow cytometry and fluorescence-activated cell sorting. In the selected soil sample, the success rate of cultured bacteria was doubled by selecting viable and metabolically active bacteria. It was determined that most of the uncultured fraction was not dormant or dead but likely required different growth conditions. It was also determined that the staining process introduced changes in the taxonomic composition of the outgrown bacterial biomass, which should be considered for further developments. This research shows the potential of flow cytometry and fluorescence-activated cell sorting applied to soil samples to improve the success rate of bacterial cultivation by estimating the proportion of dormant and yet-to-be-cultured bacteria and by directly excluding dormant cells from being inoculated into growth media.

## Introduction

It has long been known that the vast majority of bacteria within environmental samples are not culturable under standard laboratory conditions [1], understanding “cultivation” as the ability to form macroscopic colonies on standard agar media. This phenomenon was termed as the “great plate count anomaly” [2], referring to the large discrepancy between numbers of bacteria obtained by cultivation on agar plates versus direct microscopic counts. Alive bacteria that fail to be cultured generally fall into two categories: (i) their natural growth requirements have yet to be adequately fulfilled in the laboratory, or (ii) they are in a non-replicating physiological state [3, 4]. The first category constitutes the “yet-to-be-cultured” microbiota, and the most common reason for their unculturability is the failure to provide the correct biotic (e.g., nutrients and other microorganisms) or abiotic (e.g., growth signals, pH, temperature, salinity, and oxygen) factors to the growth media [3, 4]. Although the culture conditions are being elucidated for increasing numbers of environmental bacteria and other microorganisms, the use of molecular techniques has revealed that most microbiota still belong to the “yet-to-be-cultured” fraction [1, 5].

The second category of uncultured bacteria from a soil sample can be explained by physiological states in-between life and death, in which bacteria are naturally present [6]. Although there is some semantic controversy [6], bacteria in this category belong to readily culturable phylogenetic taxa but are in a “viable but not culturable” (VBNC) state [3]. Generally, VBNC cells fail to grow either because their physical integrity is compromised (i.e., they are injured, especially in their envelopes), or because they are in a “dormant” state (i.e., only grow after a resuscitation step) [3, 7, 8]. Dormant microorganisms are especially relevant in microbial ecology, as it is believed that the majority of the soil microbial biomass of most environments is in a dormant state at any specific time [7, 9]. This has been understandably ascribed to the relative low nutrient availability and the harsh environmental conditions in most ecosystems [7, 9]. It has been further explained that bacteria are constantly switching between dormancy and growth in response to environmental changes [9, 10].

Properties shown during dormancy in bacteria include, among others, reduction in cell size, change in cell shape and cell membrane composition, DNA condensation, and reduction in metabolic activity [3, 7]. The latter is considered the most defining characteristic of dormant bacteria, so it is usually measured to determine whether cells are in dormant or active states [7]. Some of the techniques to quantify metabolic activity are the measurements of ATP levels, respiratory activity, membrane potential, and heat flow; viability PCR; metaproteomics; isotope labelling; and the use of viability dyes [11, 12]. Among these methods, the use of viability dyes in combination with flow cytometry (FCM) provides a valuable tool for the characterisation of the metabolic status of bacterial cells [4, 13]. In this sense, FCM allows for a rapid and configurable high-throughput quantification of the metabolic activity level at a single-cell level through the staining of bacterial samples with specific fluorescent dyes [4, 6, 8, 13]. Furthermore, if FCM is followed by fluorescence-activated cell sorting (FACS), physical isolation of target cells can be easily performed [4]. As physical isolation of bacteria facilitates their axenic cultivation, FACS is a great tool to obtain individual colonies and pure cultures, which are still necessary to characterise bacterial representatives phenotypically [5, 14].

Several authors have made use of FCM and FACS to tackle the unculturability of environmental bacteria [15]. Notably, Zengler et al. [16] developed a technique to culture previously uncultured bacteria based on the encapsulation and incubation of cells in microdroplets, followed by detection of outgrown samples by FCM. Recently, Kurm et al. discovered that rare soil species are as likely to be dormant as abundant species [17]. On the other hand, some studies have used FCM coupled with viability dyes to demonstrate the detection of alive environmental microorganisms, which otherwise failed to grow in culture media [13, 18, 19]. Instead of traditional viability dyes, advanced techniques, such as BONCAT (bioorthogonal non-canonical amino acid tagging) [20, 21] or FISH (fluorescence in situ hybridisation) [22], have been successfully combined with FCM for the detection of protein-synthesising cells or taxon-specific bacteria, respectively. However, despite astonishing advancements in microbial ecology through genomics, metagenomics, proteomics, and bioinformatics [23], our understanding of yet-to-be-cultured and of VBNC cells in environmental microbial communities is still limited [24]. Given the recent interest in bioprospecting of the microbial dark matter in search of new bioactive metabolites [25], efforts to approach bacterial unculturability are still needed. Consequently, attempts to grow phylogenetically novel species should be complemented with approaches on the physiological requisites of reproductive viability, including the implication of states like dormancy and injury.

With that in mind, the present study first aimed to develop a procedure using FCM and FACS and the dyes cFDA and PI to select reproductively viable bacteria within soil samples for their incubation, maximizing the ratio of cultured to inoculated cells. Secondly, to explore the relative contribution of dormancy and of phylogenetic novelty on unculturability, the developed procedure was also applied to cells belonging to readily culturable taxa. Finally, to test the possible effect of the viability staining on the taxonomic composition of the culturable soil bacteria, 16S rRNA gene sequencing was performed on subsets of cultured samples.

## Methodology

### Microorganisms and growth conditions

#### Type-1 samples: Control samples

*Lactobacillus plantarum* 17–5 (ATCC 8014) [26] was purchased from American Type Culture Collection (Manassas, VA). This strain was used as a control strain to establish the optimal staining and cell sorting conditions. Cultures of *L*. *plantarum* were prepared by inoculating MRS broth with a loopful of colonies grown on an agar plate and incubating overnight in a rotary shaker at 30°C. *Escherichia coli* strain K-12 substrain MG1655 (ATCC 47076) [27] was kindly provided by Kim Lewis (Northeastern University, Boston, MA, USA). Cultures of *E*. *coli*, grown in Luria–Bertani broth (Sigma-Aldrich, St. Louis, MO, USA), were used to confirm the flow cytometry settings established with *L*. *plantarum*.

The control samples consisted of untreated and heat-treated (70°C 10 min) suspensions of 10^9^ CFU/mL of *L*. *plantarum* or *E*. *coli* in 0.1 M potassium phosphate buffer pH 7 (KPB). Finally, 1-mL sample aliquots were centrifuged (12000 x g for 10 min) to obtain a pellet of approximately 10^9^ microorganisms.

#### Type-2 samples: Extraction of soil bacteria

A soil sample was taken from the Cwmbran Boating Lake Park in the United Kingdom (51° 38’ 27.8” N, 3° 00’ 27” W). Permission for taking the soil samples was given by Torfaen County Borough Council. The sample was stored at 4 ⁰C in the dark until used (less than 2 weeks of storage time across all experiments). For the direct extraction of soil microorganisms, a vortexing treatment with extraction reagents was applied, followed by vacuum-filtration. Briefly, 20 g of soil was mixed with 40 mL of a NaCl solution adjusted to the conductivity of the soil sample. After 20 min of vortexing and 5 min of letting the soil slurry settle, 5 mL of the supernatant were vacuum-filtered through a 12-μm membrane, and the pass-through was vacuum-filtered through a 0.1-μm filter. The filter was vortexed with 5 mL of NaCl solution (previously adjusted to the conductivity of the soil sample), recovered, and centrifuged to obtain a pellet of approximately 10^9^ microorganisms.

The double-filtration step described to extract microorganisms from soil allowed for the recovery of bacteria and possibly small fungal spores. However, although no fungicide was added to the growth agar, fungal colonies were not observed under any of the assayed conditions.

#### Type-3 samples: Previously cultured soil bacteria

Bacteria were extracted from soil samples according to the aforementioned procedure, suspended in saline solution, and plated onto R2A agar plates [3, 28]. After the plates were incubated at 17°C for 4 days, the resulting grown biomass was resuspended in saline solution and centrifuged to obtain a pellet of approximately 10^9^ microorganisms.

### Staining procedures

Regarding the choice of fluorescent dyes, propidium iodide (PI) was selected as an indicator of the loss of membrane permeability [11, 12] at a concentration of 80 μM, as previously done [29]. The selected indicator of metabolic activity was 5(6)-carboxyfluorescein diacetate (cFDA, Sigma-Aldrich) because it does not impair bacterial growth after staining [28, 30, 31]. The optimal concentration of cFDA was set at 10 μM (Data in S1 Appendix). EDTA was added at 60 μM to increase the permeability of the dyes in gram-negative bacteria [6].

The staining was performed as follows: each Eppendorf containing a pellet with 10^9^ microorganisms/mL was resuspended in 1 mL of staining solution (KPB with 80 μM of PI, 10 μM of cFDA and 60 μM of EDTA) and incubated at 30°C in the dark for 30 min. For each sample, right before the FCM analysis, 10 μL were added to previously refrigerated tubes containing 1 mL of PBS with 0.8% bovine serum albumin to maximize cell viability. Samples were filtered using a 0.2-μm filter right before FCM analysis.

As an additional control measure, the PI and cFDA fluorescence of selected type-1 samples were measured with a spectrofluorometer (FLUOstar Omega, BMG Labtech, Ortenberg, Germany). The methodology for the preparation of tubes for the FCM analysis of type-2 and type-3 samples is detailed in the protocol https://dx.doi.org/10.17504/protocols.io.biazkaf6.

### Flow cytometry and cell sorting conditions

Flow cytometry (FCM) analysis and fluorescence-activated cell sorting (FACS) were carried out using a BD FACS AriaTM III (Becton Dickinson, San Jose, CA, USA). Stained and unstained cells from samples type-1 and type-3 were previously used as controls for adjusting detections and compensation settings. Compensation settings are shown in Table A in S2 Appendix. The 488-nm emission line of a 15/mW argon ion laser was the only light source used. Forward-angle light scatter and right-angle light scatter were detected with a 488/10 band-pass filter. Fluorescence emission for cFDA was detected by collection with the 530/30 band-pass filter, and fluorescence emission for PI was detected by collection with the 585/42 or the 616/23 band-pass. BD FACSFlow solution (Becton Dickinson) was used as sheath fluid. The flow rate was below 5.0 (corresponding to approximately 38 μL/min) to keep the acquisition lower than 2,000 events per second. At least 30,000 cells were acquired for analysis, and all parameters were collected at a logarithmic scale. Data was collected with the BD FACSDiva software program (version 8.0.1.) and was further analysed with FCS Express 7 Research Edition (De Novo Software, Pasadena, CA, USA).

Cell sorting was performed with the sorting application of the BD FACSDiva software, using the 85-micron nozzle. Manual gating was performed by selecting for cFDA-positive and PI-negative cells. Ungated and gated cells were sorted into microtiter plates containing MRS agar (for type-1 samples, only *L*. *plantarum*) or R2A agar (for type-2 and type-3 samples) at a concentration of one or two cells per well. Microtiter plates were incubated at 30°C for 2 days or 17°C for 4 days, and colonies were visually enumerated. FACS parameters were validated when type-1 samples of *L*. *plantarum* rendered a ratio of cultured to inoculated cells > 98% on MRS agar.

### Sequencing of stained and unstained samples

#### Sample preparation and DNA extraction

To examine the effect of the staining process on the taxonomic identity of the recovered bacterial species, sequencing results of the 16S rRNA gene microbial profiling were compared between unstained—as control—and stained samples. For this, type-2 samples were either subjected to the staining procedure or resuspended in KPB for 30 min. After incubation on R2A agar, the biomass grown on each type of plate (at least 20 colonies) was collected, and their genomic DNA was extracted using the GenElute Bacterial Genome DNA kit (Sigma-Aldrich).

#### Amplicon PCR and sequencing

Amplicon sequencing was performed using Ion Torrent technology (Thermo Fisher Scientific, Waltham, MA, USA) after amplifying the V1–V2 region of the 16S rRNA gene. This region was selected according to the manufacturer’s recommendations [32] using the primers 5’-AGAGTTTGATCMTGGCTCAG-3’ and 5’-CYNACTGCTGCCTCCCGTAG-3’, with corresponding barcode adapters from Ion Xpress (Thermo Fisher Scientific). The PCR conditions used were 5 min at 95°C, 35 cycles of 30 s at 94°C, 30 s at 55°C, and 90 s at 72°C, followed by 10 min at 72°C.

The PCR products were subjected to electrophoretic separation followed by purification with the Monarch DNA Gel Extraction kit (New England Biolabs, Ipswich, MA, USA). Template preparation was done according to the supplier’s guide (Thermo Fisher Scientific) by emulsion PCR in the Ion OneTouch2 System with the Ion PGM Template OT2 400 kit, followed by enrichment of the template-positive Ion Sphere particles. After that, a sequencing run was performed with the Ion 314 Chip v2 BC on the Ion Personal Genome Machine System employing the Ion PGM Hi-Q View OT2 kit. After completion of the run, sequences were automatically demultiplexed and filtered by the software (Torrent Suite v5.4, Thermo Fisher Scientific) to trim barcodes and adapters and to remove low-quality reads and polymorphic variants. Raw reads were exported as two fastq files: one corresponding to unstained bacteria and the other representing stained bacteria.

#### Sequence analysis

Geneious Prime 2019.1.3 (Biomatters Ltd., Auckland, NZ) was used to trim primers and to remove sequences shorter than 100 bp [33]. Taxonomic classification of the sequences was performed using the QIIME2 pipeline, v2019-4 [34]. Briefly, sequences were dereplicated into numbered amplicon sequence variants using the vsearch plugin, and taxonomy was assigned to variants using a Naïve Bayes classifier implemented in the q2-feature-classifier plugin [35]. This classifier was trained on the Greengenes 13_8 99% OTU database [36] trimmed to the V1–V2 region with the aforementioned primers. The resulting taxonomic data were imported from Qiime2 to the R v3.6.1 environment for further data analysis using the Phyloseq v1.29.0 [37] R package. Differential abundance analysis and graphic representation were performed with the R packages microbiomeSeq [38] and ggplot2 [39], respectively.

A schematic summary of the process used to perform the taxonomic classification with Qiime2 and the import operation to Phyloseq is accessible from https://dx.doi.org/10.17504/protocols.io.bh8pj9vn.

## Results and discussion

### Validation of the FCM process

FCM and FACS were used in this study to sort and inoculate, in agar, reproductively viable bacteria (i.e., cells with ability to grow macroscopically on agar to CFU levels). As reproductive growth in bacteria requires metabolic activity and envelope integrity [8], a dual staining procedure with cFDA and PI was designed based on previous studies [31], with the additional inclusion of EDTA as a permeabilising agent for gram-negative bacteria [6]. Metabolic activity is commonly assessed through cFDA, as it only fluoresces when hydrolysed by internal esterases and, therefore, is retained within the cell, while PI does not penetrate cells with intact envelopes [11, 12]. Consequently, cells with reproductive ability would be those stained with cFDA (cFDA+) but not stained with PI (PI-).

**Fig 1:**
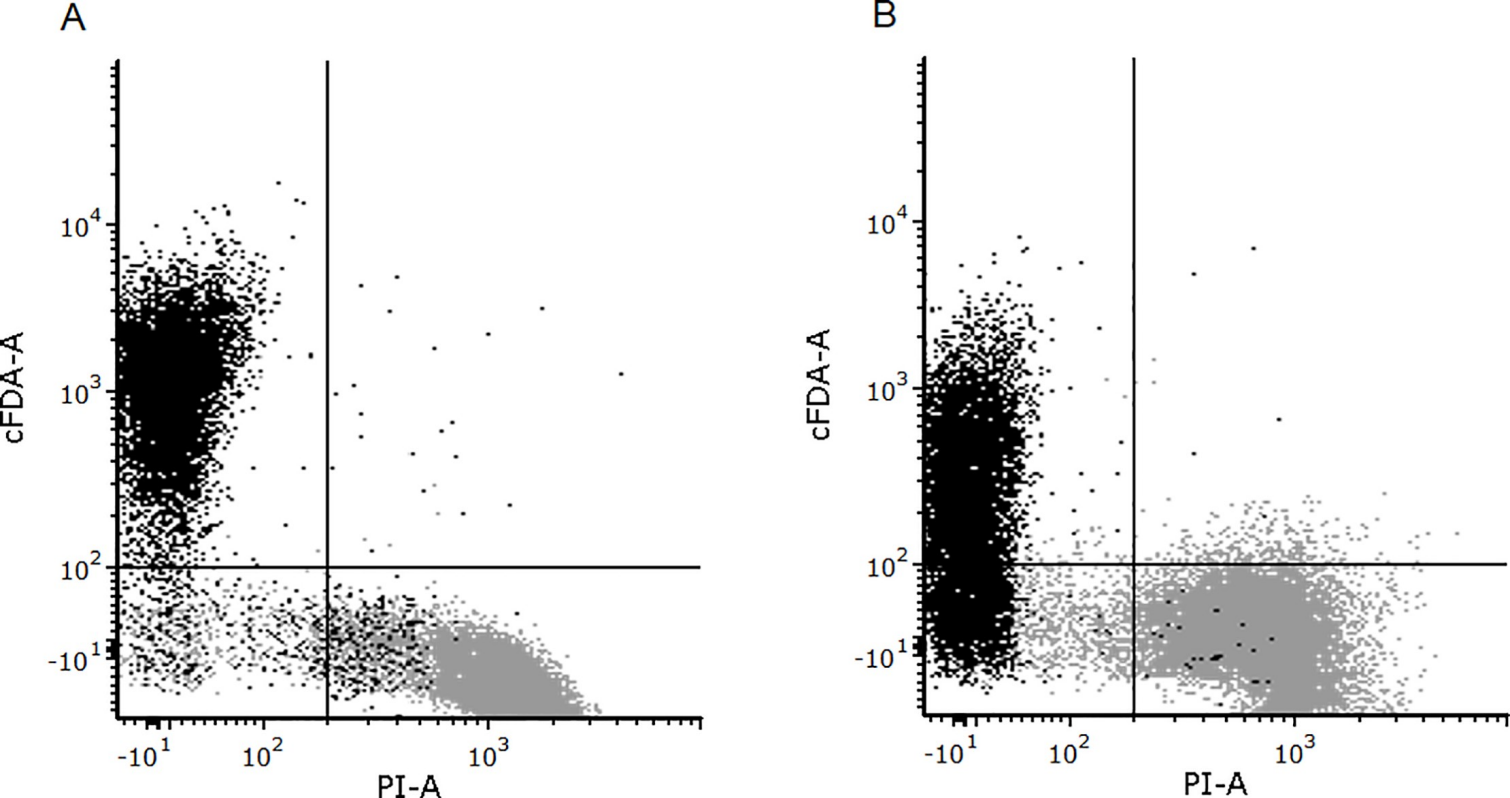
FCM of laboratory bacteria. Flow cytometry scatter plots illustrating distributions of PI fluorescence (X-axis) versus cFDA fluorescence (Y-axis) for heat-treated (dots in grey) or untreated (dots in black) stained populations of *L*. *plantarum* ATCC 8014 (A) or *Escherichia coli* ATCC 47076 (B).

Preliminary experiments carried out on type-1 samples (suspensions of *L*. *plantarum* or *E*. *coli*) confirmed that the staining procedure was not lethal (data in S1 Appendix). Type-1 samples were also employed to assess the accuracy of the staining procedure with specific FCM settings to distinguish between alive and thermally inactivated cells. A representation of the results can be seen in Fig 1, where thermally inactivated cells were used to identify the location of the quadrants. In this type of dot plot, cells identified as viable (or with reproductive ability, being cFDA+ and PI-) are located in the upper left quadrant, while inactivated cells are located in the bottom right [31]. The resulting intersection point of the quadrants is shown in Table B in S2 Appendix, and the same quadrants were used for type-2 and type-3 samples. The percentage of cells identified in each quadrant in Fig 1 is specified in Table C in S2 Appendix. This table shows that the observed numbers were closer to those expected for *L*. *plantarum* than those for *E*. *coli* for untreated and treated samples. This implied that the detection of viability was more accurate for *L*. *plantarum* than it was for *E*. *coli*, in agreement with previous results [28, 40]. This observation could be due to the leakage of the hydrolysed product of cFDA from *E*. *coli* cells and, possibly, from other gram-negative cells [28, 40]. Despite this, in the present study, more than 70% of untreated cells of either strain were categorised as viable, so the staining and FCM conditions were validated for their application in soil samples.

### Differentiation between readily culturable and yet-to-be cultured bacteria

It has been previously established that uncultured soil bacteria can either belong to yet-to-be-cultured taxa or be present in a VBNC state [3]. In the present study, the contribution of either category into the total uncultured bacteria in a soil sample was explored in a simple way. Essentially, microorganisms directly extracted from soil (named “type-2 samples”) were plated onto agar plates, and the outgrown biomass was collected after incubation (named “type-3 samples”). Consequently, type-3 samples contained only the cells from taxonomic groups able to grow on agar at the assayed conditions (referred to as “readily culturable” in this work, as opposed to “yet-to-be-cultured” taxa). Regarding their metabolic state, most cells in type-3 samples were likely viable but presented different degrees of metabolic activity, as nutrient-starved cells in the centre of bacterial colonies tend to enter dormancy [41, 42].

### Results of the FCM+FACS process

**Fig 2:**
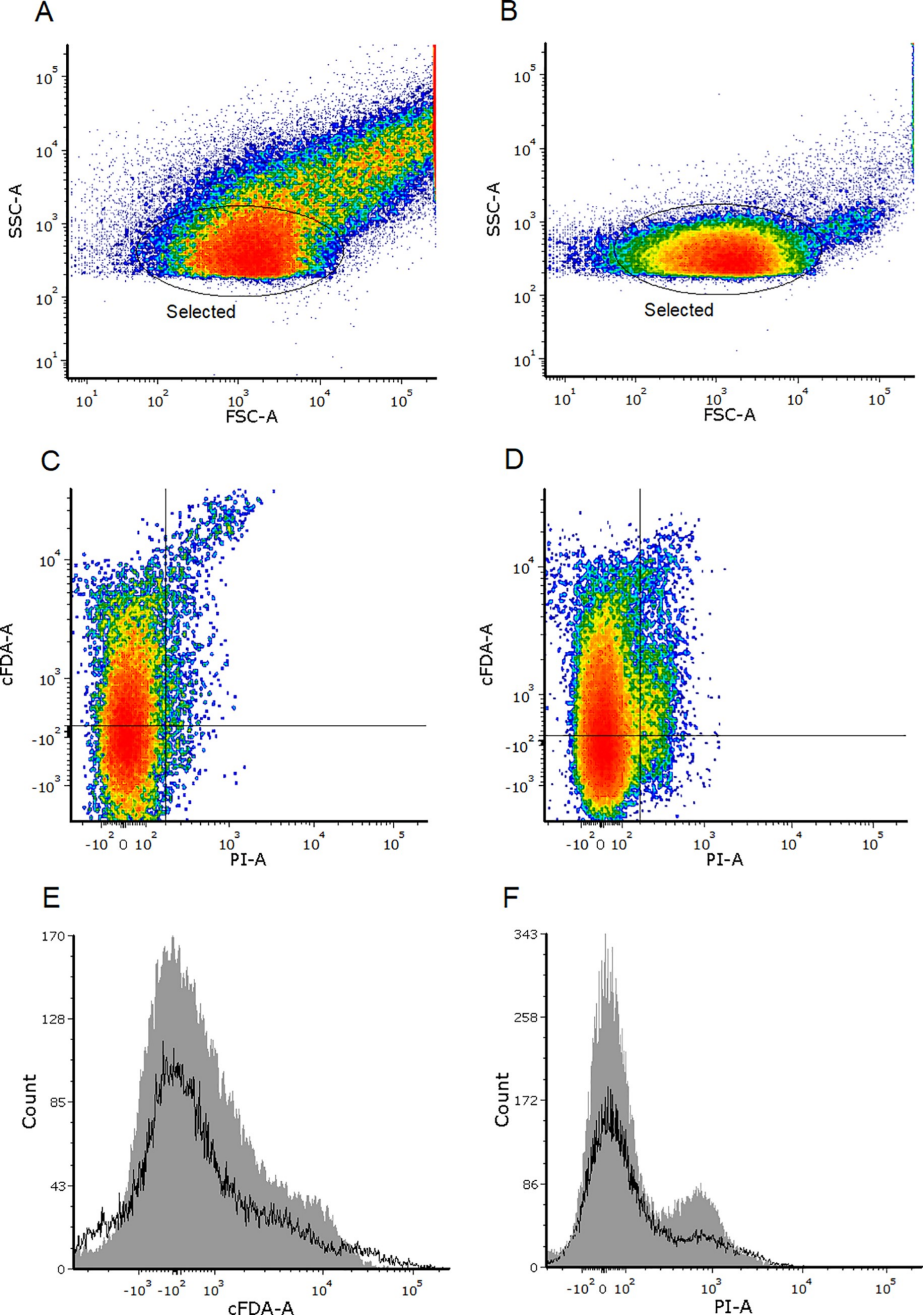
FCM of soil microorganisms. Flow cytometry plots depicting the fluorescence detected from microorganisms directly collected from soil (type-2 samples) or from biomass grown on agar plates from soil samples (type-3 samples). All samples were stained with cFDA and PI. In density plots A (type-2 samples) and B (type-3 samples), a gate was created to select a subset of events from both types of samples of similar size and complexity to analyse. The dot plots from this subset are shown in C (type-2 samples) and D (type-3 samples). The frequency distribution of the fluorescence values from this subset is shown in histograms E (for cFDA) and F (for PI); black outlined histograms represent type-2 samples and grey-filled histograms are type-3 samples. The represented data was obtained from four technical replicates for each type of sample.

Fig 2A and 2B show the different profiles of size and complexity of the events analysed by FCM from type-2 and type-3 samples, respectively. The higher heterogeneity in Fig 2A is probably due to the vast diversity of soil microbiota that is not able to grow under standard laboratory conditions [1, 5] and maybe to the presence of small contaminants derived from soil particles [6, 43].

For a pairwise comparison between both types of samples, a gate to select a cluster of intermediate size and complexity was created (Fig 2A and 2B). The fluorescence emitted by cells within this gate is shown in Fig 2C–2F. Additionally, Table D in S2 Appendix shows the percentage of cells from the selected cluster (and from the original ungated samples) belonging to each quadrant in Fig 2C and 2D. As this table and the histograms in Fig 2E and 2F show, for each dye, a very similar fluorescence pattern was observed when comparing type-2 with type-3 samples. More concretely, for both types of samples, more than 80% of events presented intact envelopes (PI < 200 fluorescence units), and about 45% of events were both PI- and cFDA+ (cFDA > 100 fluorescence units).

For the cell sorting process to inoculate in agar, a subpopulation of cells within the cFDA+ PI- quadrant was selected by manual gating. The brightest green-stained cells were excluded from this gate because they did not grow into colonies in preliminary sorting experiments. With this gating, for sorting purposes the cFDA+ PI- fraction corresponded to 33% of the bulk population.

A flow chart summarising the process and the obtained results is shown in Fig 4. Despite the similar level of metabolic activity detected in both types of samples, the proportion of cells that grew after FACS-mediated sorting was very different. About 3.5 and 66% out of the bulk of cells sorted from Fig 2A and 2B, respectively, formed colonies in agar. When selecting only the events corresponding to the non-injured and metabolically active fraction (cFDA+ and PI-), the same culturability was observed for type-3 samples (about 67%) (Fig 4). In contrast, gating for cFDA+ and PI- doubled the amount of cells that were able to grow from type-2 samples (3.5 vs. 7.3%).

In type-3 samples, about 45% of bacteria belonged to the cFDA+ PI- category (Fig 2D and Table D in S2 Appendix), while the cultured to inoculated ratio was about 67%, regardless of the metabolic state of the inoculated cells. This proportion is in agreement with the high viability of relatively fresh bacterial colonies grown on agar [42]. Possible bias in the staining by cFDA could have led to an underrepresentation of metabolically active cells in type-3 samples, as the variation coefficient in the cFDA-mediated emission (Fig 2E) was even larger than the observed variation for *E*. *coli* in Fig 1B. Hoefel et al. [13] previously described this great variability in cFDA fluorescence in environmental bacteriota, attributing it to the presence of different indigenous bacterial species with varying capacities to uptake and retain cFDA.

**Fig 3:**
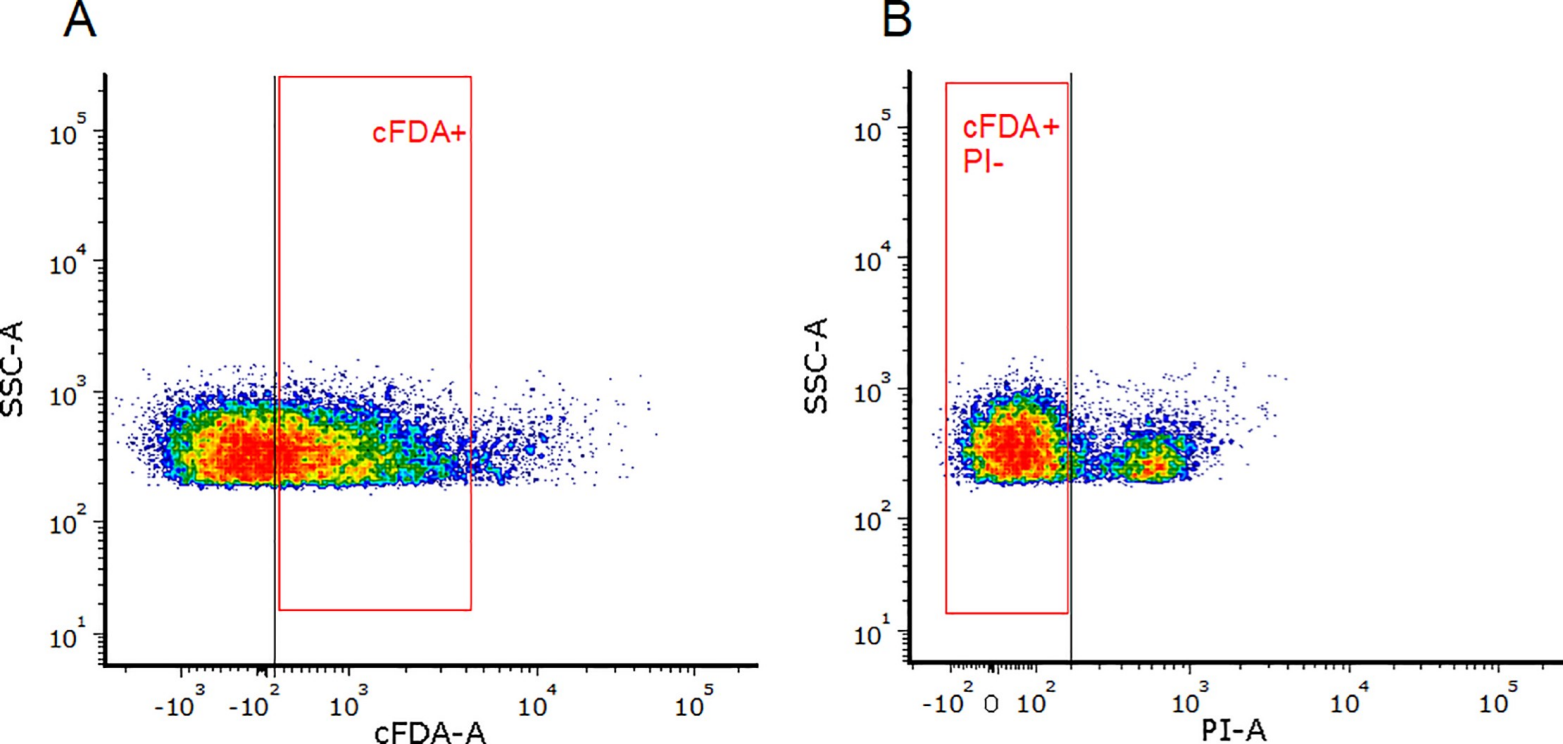
FACS of soil microorganisms. Representative flow cytometry density plots depicting the gating conditions prior to sorting. Cells were sorted into the receiving plate either from the bulk population (sorting without gating) or with manual gating to select regions that were cFDA+ and PI- (sorting of viable active cells). Example shown from a replicate of a type-3 sample.

**Fig 4:**
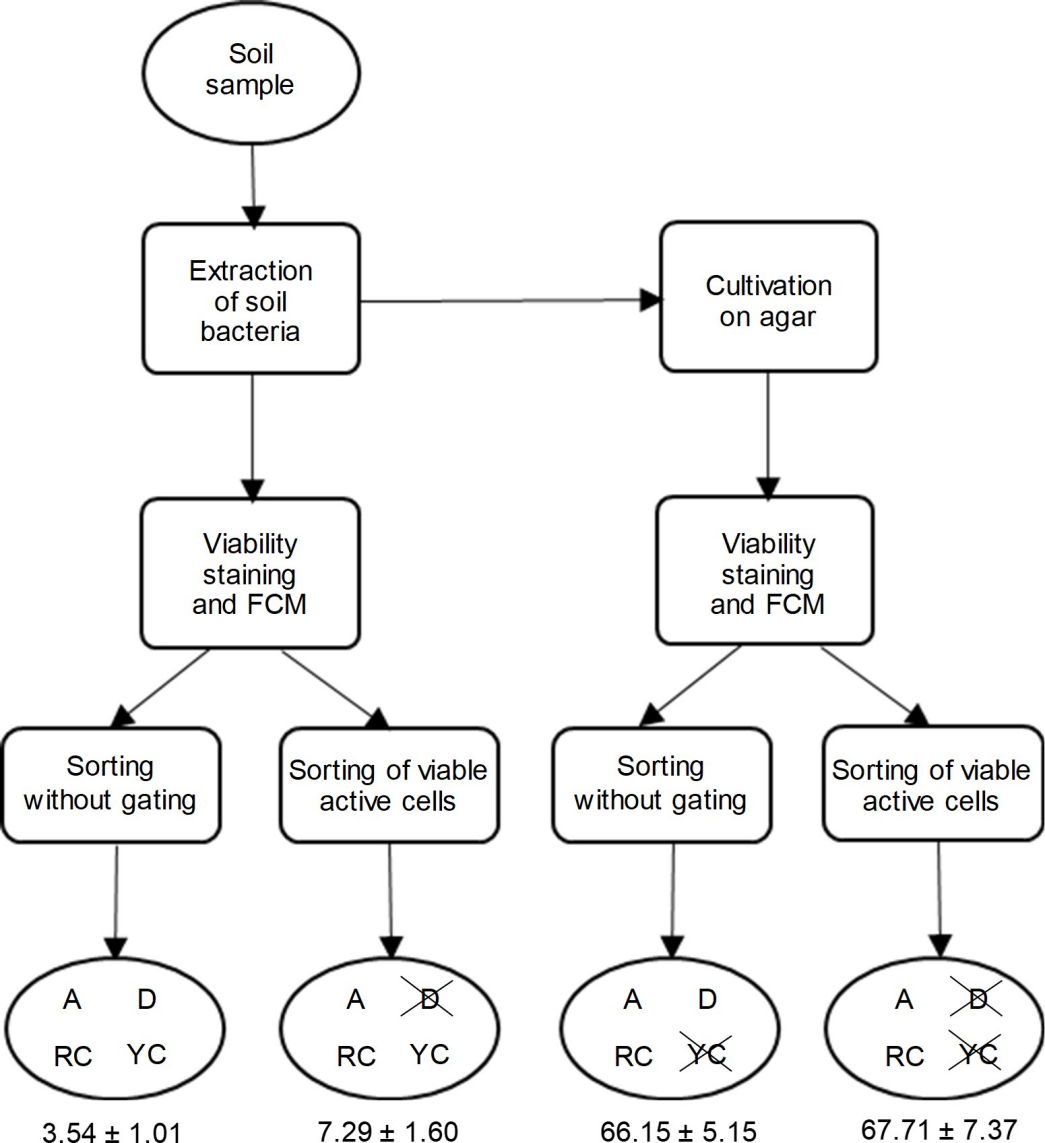
Diagram of the FCM+FACS process. Summarised flow chart of the whole procedure. The ovals show the types of cells that are automatically sorted and inoculated onto agar according to each condition. Strikethrough indicates that these types of cells are not inoculated. A = active, D = dormant and dead, RC = belonging readily culturable taxa, and YC = belonging to yet-to-be-cultured taxa. Out of all these types of cells, only those that are both A and RC are supposed to grow. For each condition, the numbers represent the percentage of grown cells (average ± standard deviation) out of the inoculated cells. Data from five type-2 samples (soil-collected bacteria) and two type-3 samples (agar-collected bacteria) from experiments performed on two different days, with 96 events inoculated on R2A agar per sample.

In comparison with type-3 samples, a similar proportion of cells (42%) in type-2 samples were considered metabolically active, according to the criterion for the cFDA+ PI- category (Fig 2C and Table D in S2 Appendix). The accuracy of this proportion is difficult to assess in terms of comparison with previous bibliography, as the metabolically active fraction of soil microbiota is highly variable according to environmental factors [10, 44] and has been estimated in broad intervals of 2–25% [2, 45] or 20–70% [20]. Independently of the proportion of metabolically active cells, in type-2 samples, the selective gating shown in Fig 3 doubled the cultured to inoculated ratio (Fig 4), which suggested the staining procedure had a positive tendency to select for metabolically active cells. The small percentage of cells that grew in agar (3.5%) is in agreement with many past studies estimating low numbers of outgrown cells in relation to total soil bacteria: from 1% [41] to 19% [46], and generally less than 5% [1, 4, 44].

Those and other studies consistently and empirically have validated the “great plate count anomaly” described by Staley and Konopka [2]. There has been increasing interest in unravelling the phenomenon of bacterial dormancy [7, 45] and the phylogenetic structure of yet-to-be-cultured microbiota [5, 12, 14]. Despite such efforts, very few works have considered and quantified VBNC cells and yet-to-be-cultured bacteria in soil. In this regard, in aquatic environments, the number of outgrown cells on agar has been compared with those of active (instead of total) bacteria [13], and this determination could be considered an estimation of the yet-to-be-cultured population. Regarding soil environments, Kurm et al. [17] recently demonstrated that rare soil bacteria do not show greater levels of dormancy than abundant bacteria and presented interesting insights into the cultivation of rare soil bacteria. In general, more work is needed to carry out similar work in soils and, especially, to adapt to soil microbiology FCM techniques that have been extensively used for aquatic environments and medical science [43].

The initial approximation into the nature of unculturable bacteria in a soil sample (schematically shown in Fig 4) could help guide, in simple steps, the best cultivation approaches in a high-throughput workflow. In the present work, two key observations resulted from the percentages shown in Fig 4. First, the culturability of type-3 samples was nearly 20 times that of type-2 samples (3.5% vs. 66%), indicating that the most crucial factor hindering bacterial growth was the presence of yet-to-be-cultured species rather than dormant or non-viable cells. However, when gating to inoculate only the metabolically active and non-injured events, the culturability of type-2 cells doubled to 7% (Fig 4). The increased ratio in cultured to inoculated microorganisms after FACS has been achieved in other works [8] but usually employing cultures grown in vitro instead of natural microbial communities [12]. From a practical point of view, environmental variations heavily influence the level of dormancy in bacterial communities [7, 10], so its estimation can help design the proper treatment for each soil sample (e.g., intensive resuscitation steps if a high proportion of cells are in the VBNC state) and, therefore, increase the success rate of the cultivation process. Overall, the positive preliminary results and conceptual idea presented in Fig 4 could lead to the development of a simple procedure to increase the culturability of soil bacteria.

### Bias introduced by the staining process

The phylogenetic diversity and physiological heterogeneity of cells within a soil sample represent a challenge for the success of FCM and FACS-based techniques [12]. For this reason, more research is necessary to improve the whole procedure by tackling each one of the stages summarised in Fig 4 (i.e., bacterial extraction from soil, viability staining, FCM analysis, FACS-based inoculation, and cultivation in growth media). In this respect, high-throughput sequencing technologies have become an invaluable tool in the characterisation of environmental microbial communities by providing insight into their taxonomic identities and relative abundance [20]. As they are culture-independent methodologies, sequencing techniques are crucial not only to broaden our knowledge of yet-to-be-cultured taxa [4, 5, 20] but also for coupling with other technologies, such as FCM, to resolve existing challenges and develop new procedures [47]. With regard to the present work, sequencing of the bacterial content at different stages (such as before FCM or after FACS) would help choose methodological conditions to refine the whole procedure.

Among all steps, the staining procedure has an enormous impact on FCM outcomes [48], and extensive guidance has been published on the discriminatory power and applications of different stains [12, 15]. Nevertheless, little is known about how distinct affinities of stains towards structural and physiological characteristics of certain taxa can change the taxonomic profiling of the samples during preprocessing or FCM analysis. Based on this idea, the present work aimed to explore the impact of the viability staining procedure on the taxonomic profiling of readily culturable bacteria through the compared 16S rRNA gene sequencing of unstained and stained samples.

**Fig 5:**
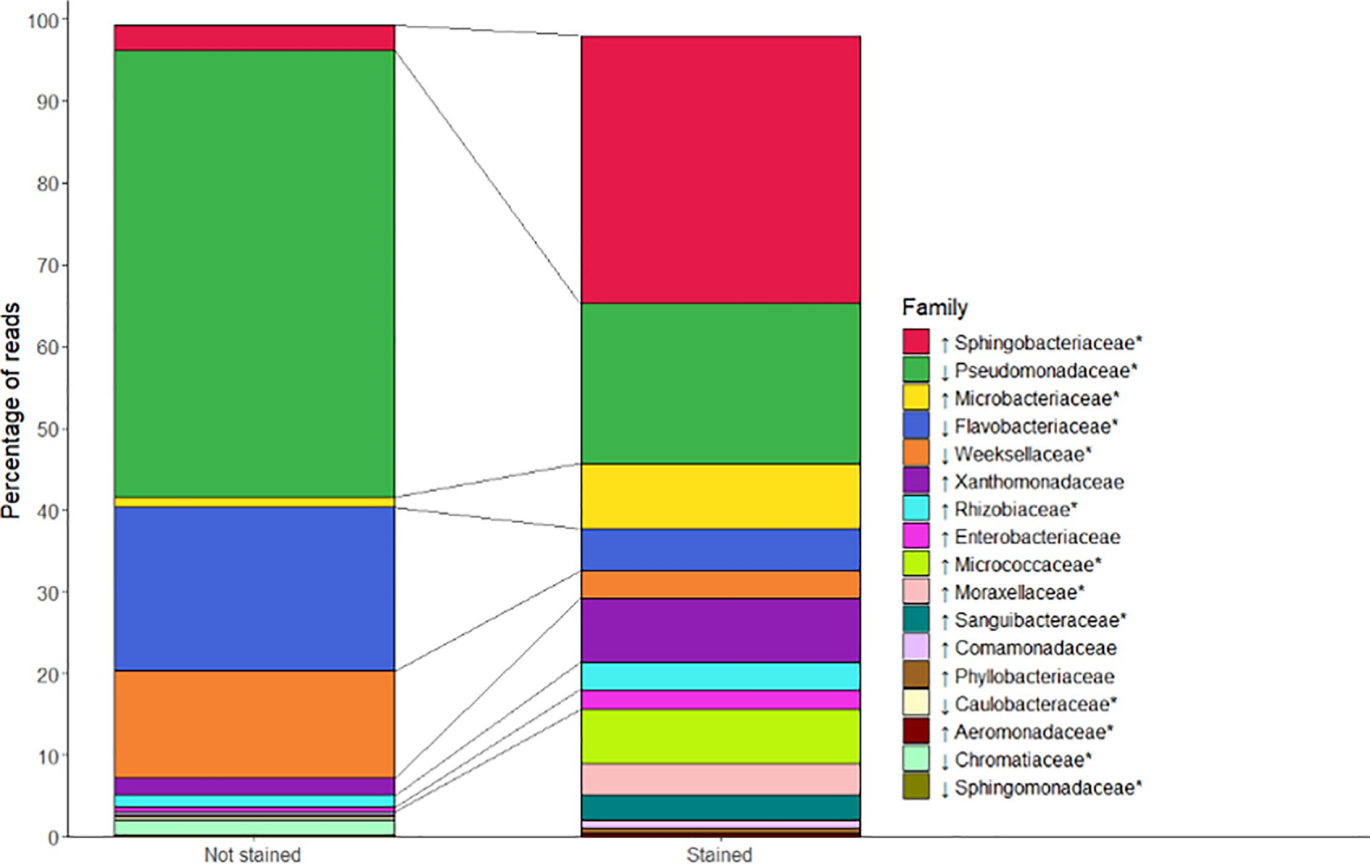
Change in taxonomic families due to the staining process. Stacked bar plots showing the relative abundances of bacterial families of a sample subjected to a viability staining process (cFDA, PI, and EDTA) compared to those of an unstained sample. Bacteria directly extracted from soil were either subjected to a control process (Not Stained) or to the staining process (Stained) and plated onto agar before DNA extraction for 16S rRNA gene sequencing. Arrows represent increase or decrease in the relative abundance of the family in the stained sample in comparison with that of the unstained sample. Asterisks represent statistically significant (*p* < 0.05) changes.

Fig 5 shows a summary of the phylogenetic analysis at the family level, containing the relative abundance of families present with a higher than 0.15% average abundance across both samples. In the unstained sample, the most prevalent outgrown families were *Pseudomonadaceae* (54.6% of relative abundance), *Flavobacteriaceae* (20%), *Weeksellaceae* (13%), and *Sphingobacteriaceae* (3.2%). Within each of these families, nearly all the sequencing reads belonged to the genera *Pseudomonas*, *Flavobacterium*, *Chryseobacterium*, and *Pedobacter*, respectively. It should be noted that the aforementioned taxonomic groups are among the most common readily culturable soil bacteria (*Pseudomonas*, *Flavobacterium*, and *Pedobacter*) [49] and have been found in soil rich in hydrocarbon pollutants (*Pseudomonadaceae*, *Flavobacteriaceae*, and *Weeksellaceae*) [50]. This taxonomic profiling seemed consistent with the environmental history of the soil, as the sample was obtained from a site notorious for its past coal and oil mining industry [personal communication].

As shown in Fig 5, subjecting a type-2 sample to the staining process led to a substantial change in the taxonomic composition of the outgrown bacterial mass. The relative abundance of the three most prevalent families in the unstained sample decreased significantly after staining, but this reduction was compensated by an increase in the relative abundance of other groups, including the Actinomycetales *Micrococcaceae* and *Microbacteriaceae*. However, the major increase was in the genus *Sphingobacterium* (within the family *Sphingobacteriaceae*), with a relative abundance of 29% in the stained sample versus 0.33% in the unstained sample. The proportion of the other genus present from this family—*Pedobacter*—did not change significantly.

Further research would be needed to elucidate the reasons behind the differences found in the taxonomic composition of both bacterial samples. A possible explanation could lie in the subjection of the stained samples to mild stress conditions, namely an increase in temperature and the presence of a low concentration of EDTA. In this sense, bacteria react quickly to environmental stresses and cues (such as changes in temperature, pH, osmolarity, or nutrient content) by modifying their gene expression levels, cell morphologies, and compositions [20, 51]. Some of these modifications are known to induce VBNC states, especially changes in the composition of proteins or fatty acids in cell envelopes [51]. A peculiarity of the genus *Sphingobacterium* lies on the presence of high concentrations of sphingophospholipids in cellular lipid components [52], so the staining process could have favoured the growth of bacteria with this cellular configuration, hindered the growth, or induced VBNC states in others. In addition, the staining process led to an increase in the biodiversity of the samples, as measured by Shannon’s index at the family level (1.45 in the unstained sample vs. 2.19 in stained sample), which could be a positive feature from a practical point of view. Some studies have demonstrated the effect of environmental and experimental stresses on the taxonomic composition or the diversity of bacterial communities (see [47] as an example), but little is known about the possible bias in the taxonomic profiling caused by the staining process. In addition, other manifestations of the influence of the staining process should be carefully considered when performing cell sorting. For example, a possible hidden link between specific bacterial taxa and their tendencies to be stained as cFDA+ PI- (independent of the metabolic state of the cell) could influence the proportions of outgrown cells shown in Fig 4, and therefore should be discarded in future experiments.

Overall, this finding represents a starting point for further investigation on the impact of the viability staining, among numerous factors, on the development of a FCM protocol to increase the success rate of soil bacteria cultivation. For example, in case there was an unsolvable bias in the taxonomic representation of the growing bacteria, the viability staining process could be improved by substituting cFDA for rhodamine 123 or PI for TO-PRO-3 [6, 8].

## Conclusions and further work

This work focused on the development of a simple procedure to try to increase the success rate of soil bacteria cultivation with the use of flow cytometry (FCM) and fluorescence-activated cell sorting (FACS) (summary in Fig 4). The most relevant basis for the development of this procedure was the acknowledgment of the two major causes of bacterial unculturability: the presence of cells in a viable but not culturable state and the presence of cells belonging to yet-to-be-cultured taxa. The estimation of the proportion of bacteria belonging to each group would help characterise the bacteria from the analysed soil sample in terms of unculturability, so that proper measures can be taken to increase the proportion of outgrown cells. In the selected soil sample, the experiments conducted showed that (i) the success rate of cultured bacteria could be doubled by selecting viable and active bacteria and (ii) the major reason for the lack of growth of most bacteria was that they needed more specific experimental growth conditions. Improvements in the experimental conditions are necessary, as shown by the taxonomic profiling modification of the outgrown bacteria caused by the staining process. Moreover, the substitution of the traditional dyes cFDA and PI for more advanced markers of cellular activity, such as BONCAT [20, 21], would probably help overcome the bias associated with these traditional dyes and provide a better segregation between metabolically active and inactive cells. This research shows the potential of FCM and FACS applied to soil samples to improve the success rate of bacterial cultivation by excluding dormant cells and, at the same time, pondering the need for different culture conditions.

## Supporting information

S1 Appendix Fluorescence absorbance values and CFU counts supporting the validity of the staining process. (DOCX)

S2 Appendix Tables containing supplemental information about the FCM parameters and results. (DOCX)
