# Supplementary material for: An approach to increase the success rate of cultivation of soil bacteria based on fluorescence-activated cell sorting
Source: PLoS One. 2020 Aug 31;15(8):e0237748. doi: 10.1371/journal.pone.0237748 (PMC7458294; doi:10.1371/journal.pone.0237748)
Supplement: S1 Appendix — (DOCX) [file pone.0237748.s001.docx]

**S1 Appendix. Fluorescence absorbance values and CFU counts supporting the validity of the staining process.**

Cultures of *L. plantarum* (*Lactobacillus plantarum* ATCC 8014) or *E. coli* (*Escherichia coli* ATCC 47076) were prepared at 10^9^ CFU∙mL^-1^ in 0.1 M potassium phosphate buffer, with pH 7. These suspensions were either non-treated (Control) or heat treated at 73 °C 10 min (HT), before or after the addition of stains (cFDA only; cFDA and EDTA simultaneously; or cFDA, PI, and EDTA simultaneously). If present, cFDA, PI, or EDTA were added at 10, 80, or 60 µM, respectively. The fluorometric emission of the suspensions was measured in a FLUOstar Omega microplate reader with 485-12 and 510-10 excitation and emission filters, respectively. The bacterial concentration of the suspensions of *L. plantarum* or *E. coli* was measured by colony counts on MRS agar or R2A agar plates, respectively.

|  | **Fluorescence** | | **Log CFU∙mL^-1^** | |
| --- | --- | --- | --- | --- |
|  | ***L. plantarum*** | ***E. coli*** | ***L. plantarum*** | ***E. coli*** |
| **Control** |  |  | 9.05 | 8.54 |
| **cFDA** | 42584 | 20373 |  |  |
| **cFDA & EDTA** |  | 34219 |  |  |
| **cFDA + HT** | 4731 | 3733 |  |  |
| **cFDA & EDTA + HT** |  | 2176 |  |  |
| **HT + cFDA** | 3582 |  |  |  |
| **cFDA & PI & EDTA** |  |  | 8.60 | 8.47 |
