# Supplementary material for: An approach to increase the success rate of cultivation of soil bacteria based on fluorescence-activated cell sorting
Source: PLoS One. 2020 Aug 31;15(8):e0237748. doi: 10.1371/journal.pone.0237748 (PMC7458294; doi:10.1371/journal.pone.0237748)
Supplement: S2 Appendix — (DOCX) [file pone.0237748.s002.docx]

**S2 Appendix. Tables containing supplemental information about the FCM parameters and results.**

|  | **cFDA** | **PI** |
| --- | --- | --- |
| **cFDA** | 1.00629 | -0.12579 |
| **PI** | -0.05031 | 1.00629 |

**Table A. Automatic compensation matrices for the files present in Figs 1, 2 and 3.**

**Table B. Position of the intersection point of the quadrants in Figs 1 and 2C-2D.** These quadrants were established according to the results from type-1 samples and used to determine the proportions of positively and negatively stained cells with cFDA and PI for type-2 and type-3 samples.

|  | **X Value (PI-A)** | **Y Value (cFDA-A)** |
| --- | --- | --- |
| **Center** | 200 | 100 |

**Table C. Percentage of cells identified in each quadrant in Fig 1.** The theoretical percentage is shown in parentheses.

|  | **Untreated *L. plantarum* (Fig 1A)** | | **Heat-treated *L. plantarum* (Fig 1A)** | | **Untreated *E. coli***  **(Fig 1B)** | | **Heat-treated *E. coli* (Fig 1B)** | |
| --- | --- | --- | --- | --- | --- | --- | --- | --- |
|  | **PI-** | **PI+** | **PI-** | **PI+** | **cFDA** | **PI** | **cFDA** | **PI** |
| **cFDA+** | 88.61 (100) | 0.20 (0) | 0.13 (0) | 0.04 (0) | 74.80 (100) | 0.04 (0) | 0.31 (0) | 1.89 (0) |
| **cFDA-** | 4.85 (0) | 6.34 (0) | 4.08 (0) | 95.75 (100) | 24.98 (0) | 0.18 (0) | 5.47 (0) | 92.33 (100) |

**Table D. Percentage of cells identified in each quadrant in Figs 2C and 2D, and comparison with their ungated counterparts.** No significant differences were found when comparing the four types of samples (ungated and gated type-2 and type-3 samples). For these statistical comparisons, Kruskal-Wallis tests were performed at a 95% confidence level, using four replicates for each sample.

|  | **Type-2 samples** | | | | **Type-3 samples** | | | |
| --- | --- | --- | --- | --- | --- | --- | --- | --- |
|  | **Selected (Fig 2C)** | | **Ungated** | | **Selected (Fig 2D)** | | **Ungated** | |
|  | **PI-** | **PI+** | **PI-** | **PI+** | **PI-** | **PI+** | **PI-** | **PI+** |
| **cFDA+** | 42.38 | 13.40 | 38.19 | 17.99 | 45.86 | 11.79 | 44.73 | 12.89 |
| **cFDA-** | 39.97 | 4.25 | 37.84 | 5.98 | 37.13 | 5.22 | 36.40 | 5.99 |
